# Climate Action for (My) Children

**DOI:** 10.1007/s10640-021-00620-7

**Published:** 2021-11-16

**Authors:** Helena Fornwagner, Oliver P. Hauser

**Affiliations:** 1 Department of Economics and Econometrics, University of Regensburg, Universitätsstr. 31, 93040 Regensburg, Germany; 2 Department of Economics, University of Exeter, Rennes Drive, Exeter, EX4 4PU UK

**Keywords:** Children, Intergenerational cooperation, Lab-in-the-field experiment, Observability, Parents, Voluntary climate action

## Abstract

How do we motivate cooperation across the generations—between parents and children? Here we study voluntary climate action (VCA), which is costly to today’s decision-makers but essential to enable sustainable living for future generations. We predict that “offspring observability” is critical: parents will be more likely to invest in VCA when their own offspring observes their action, whereas when adults or genetically unrelated children observe them, the effect will be smaller. In a large-scale lab-in-the-field experiment, we observe a remarkable magnitude of VCA: parents invest 82% of their 69€ endowment into VCA, resulting in almost 14,000 real trees being planted. Parents’ VCA varies across conditions, with the largest treatment effect occurring when a parent’s own child is the observer. In subgroup analyses, we find that larger treatment effects occur among parents with a high school diploma. Moreover, VCA for parents who believe in climate change is most affected by the presence of their own child. In contrast, VCA of climate change skeptical parents is most influenced by the presence of children to whom they are not related. Our findings have implications for policy-makers interested in designing programs to encourage voluntary climate action and sustaining intergenerational public goods.

## Introduction

Individual actions—referred to as voluntary climate action (VCA)—are needed to reduce climate change’s harmful effects (Goeschl et al. 2020). VCA takes different forms on an individual level; however, one key unifying aspect of VCAs is that they necessitate incur a cost to the individual to provide a benefit to the environment, a general public good that is largely consumed in the future (Fischer et al. 2004; Diederich and Goeschl 2014; Hauser et al. 2014; Lohse and Waichman 2020). Examples of VCAs include investing in energy-saving technology (e.g., solar panels), switching to CO2-friendly purchasing habits (e.g., buying less red meat), or even engaging in small, everyday behaviors, such as spending less time in the shower (Wynes and Nicholas 2017). In our study, we are interested in VCA that has a long-lasting positive effect on the environment (Layton and Levine 2003; Steinke and Trautmann 2021): we focus on CO2 offsetting, using a foresting program that plants climate-efficient trees, as such programs have become increasingly widespread and available as means for individuals to help reduce their “carbon footprint” (Kollmuss et al. 2010).

While past research has examined contextual changes (“nudges”) to motivate VCAs (Thaler and Sunstein 2008; Hauser et al. 2018) (see Appendix C for related literature), we propose a novel perspective on how to solve VCA dilemmas by leveraging the intergenerational aspect of VCA. Extant research has focused on public goods within the same generation (Fehr and Gächter 2000; Milinski et al. 2006; Rand et al. 2009) or cooperation between different generations (Charness and Villeval 2009), whereas the literature on intergenerational goods, where future generations cannot reciprocate the actions of the acting current generation and the incentives to cooperate with the future are low, is still in its early stages (Andre et al. 2021; Fischer et al. 2004; Sutter et al. 2013; Hauser et al. 2014; Kamijo et al. 2017; Ponte et al. 2017; Shahrier et al. 2017; Dengler et al. 2018).

However, this does not imply that there exists no link to future generations: people (i.e. parents) who have children are genetically related to the next generation and have an incentive and responsibility to care for their offspring’s wellbeing. Parents make up a large fraction of the population (Eurostat 2017) and, as they are in their adult life stage, they are among the largest contributors to CO2 emissions through their consumption, production, and work (Zagheni 2011). Thus, getting parents to engage in any kind of VCA is likely to result in economically meaningful changes.While parents “in isolation” have not been found to be more willing to give to VCA than other groups (Diederich and Goeschl 2014), we argue that, when their own child observes their VCA decision, the personal genetic link to the future makes them more likely to engage in VCA, as the intergenerational benefits of VCA are more salient to them.

Specifically, we predict that parents will be especially likely to engage in VCA when observed by their offspring relative to other observers. While past work has shown the importance of observers to motivate costly cooperative behaviors (Yoeli et al. 2013; Hauser et al. 2016), a parents’ offspring is critical here because it lets them recall their genetic link to the future. Therefore parents, who have their children’s wellbeing at heart, are reminded of the benefits of investing in the future when their genetic beneficiaries are present (Smith 1977; Nowak 2006).

## Methods

### Voluntary Climate Action and Study Context

We carried out a novel lab-in-the-field experiment with 368 participants in Innsbruck, Austria (for details regarding sample size, pre-registration, and power calculation, see Appendix D). The experiment included an incentive-compatible survey programmed in oTree (Chen et al. 2016), and data were collected with tablets (see Appendix E). Participation took no longer than 20 min, and participants were randomly assigned to a treatment when they were handed a tablet. Using a recruitment stand in public spaces, we recruited parents who were accompanied by at least one of their own children aged between 7 and 14 years. At all times during the experiment, only one parent (the DM) and one of the parent’s own children (who is an observer in one condition and not involved in the experiment in the other conditions) were allowed to participate. In conditions where the child was not an observer, s/he was asked to wait outside the study booth and participate in various games and activities (supervised by research assistants). In addition, for our conditions with observers who are not related to the participant, we employed confederate adults and confederate children who were introduced to the participant as “helpers from the community” to act as observers.

The VCA in our study was carefully designed based on the extant literature. It is worth noting that VCAs are generally undersupplied, and previous attempts to get participants to invest in VCA have generally not resulted in large effects; knowing this, we, therefore, aimed to design an attractive VCA for the participants in our study by incorporating some of the “best practices” from past literature. For example, past work has found that the general public prefers investing in VCA with local mitigation goals (Torres et al. 2015). Thus, in our setting, the VCA to offset CO2 takes the form of a local foresting program, for which we collaborated with the forestry office Innsbruck (“Amt für Wald und Natur” of the city of Innsbruck). We chose a foresting program for forest restoration because such programs are among the best climate change solutions available today (Bastin et al. 2019). Participants were asked to choose between keeping money for themselves or spending that money on planting trees. All trees that participants decided to plant will be planted in 2020 and 2021 on the “Nordkette” and “Patscherkofel” mountain ranges close to Innsbruck, ensuring that the mitigation strategy is truly local. Moreover, this particular area has a high suitability for the VCA, as it has a high net plant productivity with the potential for forest restoration (Bastin et al. 2019).

Following Goeschl et al. (2020), subjects received a short and neutral description of the foresting program. In particular, they were informed that the foresting program has the following characteristics: (a) The trees would only be planted if participants in our study actually chose to spend their money on planting a tree. This ensured that the participants’ decision was incentive-compatible and truly contributed to reducing CO2 in the environment. (b) The trees were selected to lead to a climate-friendly mixed forest, including climate-efficient species of different fir trees or deciduous trees. These tree types would usually not be planted as frequently due to their cost. (c) Each tree has an expected minimum age of 120 years (estimate provided by the forestry office Innsbruck). This means that each tree our participants planted lasts at least the equivalent of four average (human) generations (following the Cambridge dictionary definition of a “generation”). (d) The trees would be monitored and controlled annually to ensure they are healthy, and they would be listed in the governmental forest database “Walddatenbank” to ensure a “paper trail” of the planting exists. (e) The trees would be planted in a forest that is certified with an internationally recognized “Program for the Endorsement of Forest Certification” (PEFC) certificate, ensuring environmental sustainability. All these characteristics ensured the maximally possible credibility of our CO2 offsetting program. More information on the foresting program can be found in Appendix F.

Moreover, as part of the instructions, subjects were given information about greenhouse gas emissions and trees’ role for CO2 reductions before deciding on the VCA. Since the general population has relatively little prior knowledge about VCAs (Diederich and Goeschl 2014), we ensured that all participants first gained a basic understanding of the VCA in this study and correctly answered several comprehension questions. Whereas MacKerron et al. (2009), Löschel et al. (2013), and Goeschl et al. (2020) provided information as text on the screen, our study participants watched a short video. The video informed participants about the public goods character of CO2 reductions by explaining how planting trees removes CO2 from the atmosphere and mitigates global climate change. In particular, the video highlighted that reducing CO2 has an impact not only on current generations but also on future generations.

### Experimental Conditions

We implemented four conditions in a between-subjects design, varying observability and the type of observer (see Table 1). In all conditions, a parent received a windfall endowment of 69€ and was asked to decide how much of that money to keep for themselves and how much to invest into the VCA (i.e., planting trees). Using their endowment, participants could purchase between 0 and 46 trees, with each tree costing 1.50€ (the average cost of planting a tree in the foresting program). Any money not invested in planting trees was paid to participants in cash at the end of the experiment.

**Table 1:** Experimental conditions, varying who observes the participant

Condition	Observer	Intergenerational link?	Genetic link?
*NoObserver*	No observer	No	No
*StrangerAdult*	Adult (not related to DM)	No	No
*StrangerAdult*	Child (not related to DM)	Yes	No
*OwnChild*	DM’s own child	Yes	Yes

When making their decision, participants had detailed information on how much CO2 emissions a tree would offset every year (0.015 tons) and that the total possible amount of 46 trees would offset 10% of the average CO2 emissions of a person living in Austria (OECD 2018). Based on these numbers, the price to offset one ton of CO2 emissions per year with our VCA is fixed at 100€/t (see Diederich and Goeschl 2014 who argue that this price reflects “an economically meaningful maximum abatement cost for one ton of CO2 emissions”, see also Tol 1999, 2009, 2010), which is higher than the EUA (EU ETS) Future Price for one ton of over 60€/t (as of September 2021). We also collected data on basic demographics (e.g., gender, age, education, etc.) and included a short survey at the end of the experiment (see Appendix E).

In our baseline *NoObserver* condition, the DM decided in private without being observed by anyone. In the *StrangerAdult* condition, the DM was observed by another adult who is a hired actor (confederate) to act as the observer and who is unrelated to the DM (see detailed information about the observability procedure in Appendix E). This condition is similar to the standard procedure used in observability experiments in the lab, where a DM is observed by another adult, which helps establish a “general observability” effect. In the *StrangerChild* condition, the observer was an actor who is a child between 7 and 14 years old and unrelated to the DM, which helps identify whether the VCA can be encouraged by having an observer from the future (beneficiary) generation. Finally, in the *OwnChild* condition, the observer was the DM’s child, enabling us to study whether the DM’s *own* child uniquely affects the DM’s VCA behavior.

All observers were provided the same information as the DMs, including information about greenhouse gas emissions and the trees’ role for CO2 reductions. They also read the description of the local CO2 mitigation program. All this information is common knowledge to the DM and the observer, as both DM and observer read the instructions simultaneously during the experiment. Detailed information on the experimental design can be found in Appendix E.

### Experimental Sample

Our experiment involved 368 parents, 92 in each of the four treatment conditions. Data were collected starting at the end of 2019 until early 2020 in three different locations in the city of Innsbruck. In Appendix Table 10, we provide background details on our participants based on the post-experimental questionnaire. In Appendix Table 11, descriptive statistics are further broken down by treatment, showing that randomization worked: the randomly assigned participants are comparable across a number of relevant characteristics.

Across all treatments, 67% of our participants are female (248 out of 368), and the average age is 42 years. Participants have, on average, 2.06 children, and the vast majority (96%) are currently employed. With regards to education, 86% received a high school diploma (by completing an exam called “Matura”), which provides general access to higher education and labor market qualifications. Out of those with a high school diploma, half (50%) have a university degree. The majority is married or in a registered relationship (66%), and there is approximately an equal split between those living in the city (49%) versus those living in rural areas. Our recruited sample is largely representative of the general population of Innsbruck (Austria), where our trial took place.

Following Goeschl et al. (2020), we included a survey question asking participants how risk-seeking they viewed themselves (based on Falk et al. 2018). The mean reported value was 5.35 on a scale from 0 (not risk-seeking at all) to 10 (fully risk-seeking) and did not differ between treatments (Kruskal–Wallis test, *p* = 0.255). Additionally, we asked participants how patient they believe they are as a proxy of their time preferences. The average reported score was 5.92, measured on a scale from 0 (not at all patient) to 10 (fully patient). We did not find any treatment differences for the patience measure (Kruskal–Wallis test, *p* = 0.397).

In three out of four treatments, the participant was observed, for which we provide summary statistics of the different observer characteristics. Stranger adult observers (who were hired by the experimenters as confederates) in the *StrangerAdult* condition were on average 39.89 years old and observing children 11.33 years (*StrangerChild*: 12.23 years; *OwnChild*: 10.43 years). The majority (55%) of observers was female, and the number of female observers did not differ across treatments (Fisher’s exact *p* = 0.231). In Appendix Table 15, we summarize the gender matches of participants and observers for the treatments with observers. Because both the participant sample and the observers were made up of more women (F) than men (M), we have 99 FF matches, 88 FM, 54 MF, and 35 MM matches. Gender matches were balanced across treatment conditions (χ^2^, *p* = 0.497).

### Econometric Specifications

Our analytical strategy is twofold: First, we estimate the treatment effects on VCA using ordinary least squares (OLS) regressions, shown in columns (1) and (2) in all our regression tables. Second, we employ Tobit regressions, which can be found in columns (3) and (4) in the regression tables, to estimate treatment effects, taking into account that the dependent variable is the number of trees planted (i.e., VCA), which is bounded by 0 trees on the lower end (if the participant keeps the entire endowment for him/herself) and by 46 trees on the upper end (if the participant invests the entire endowment into the VCA). For both models, we use the following specifications for columns (1) and (3), which shows the main effects of the independent variables (treatment dummies) without any control variables:

1 $$ VCA_{i} = \beta_{0} + \beta_{1} StrangerAdult_{i} + \beta_{2} StrangerChild_{i} + \beta_{3} OwnChild_{i} + \varepsilon_{i} $$

where *i* = 1, …, n indicates participant *i*, *VCA* is a continuous variable (ranging from 0 to 46) measuring the number of trees a participant decided to plant, and the *StrangerAdult*, *StrangerChild*, and *OwnChild* dummies are 1 in the respective treatments and 0 otherwise, ${\upvarepsilon }_{{\text{i}}}$ measures unobserved scalar random variables (errors).

We also report in columns (2) and (4) the same specification with several control variables:

2 $$ \begin{aligned}   VCA_{i} ~ =  & ~\,\beta _{0}  + ~\beta _{1} StrangerAdult_{i}  + ~\beta _{2} StrangerChild_{i}  + ~\beta _{3} OwnChild_{i}  + \beta _{4} Location_{i}  \\     &  + \beta _{5} Age_{i}  + \beta _{6} Female_{i}  + \beta _{7} NrKids_{i}  + \beta _{8} Risk_{i}  + \beta _{9} Patience_{i}  \\     &  + \beta _{{10}} HighSchoolDipl_{i}  + \beta _{{11}} Employed_{i}  + \beta _{{12}} Rural + \varepsilon _{i}  \\  \end{aligned}  $$

where *Location* is a categorical variable controlling for the three study locations Rathausgalerien, Sillpark, and Herbstmesse, *Age* is a continuous variable and *Female* a dummy variable for the participant’s age and gender, *NrKids* is a continuous variable capturing the participant’s number of kids, *Risk* and *Patience* are self-reported scale measures (scale range from 0 to 10), *High School Dipl.* is a dummy variable which is 1 if the participant completed secondary education (“Matura”), *Employed* is a dummy variable which is 1 if the participant is currently employed and *Rural* is a dummy variable which is 1 if the participant lives outside a city; all other variables are as defined in Eq. (1).

## Results

### Descriptive Results

For the 368 participants in our study (see Appendix Tables 7, 8 and 9 for descriptive statistics), we find a remarkable willingness to engage in VCA: across all conditions, all participants combined chose to plant a total amount of 13,988 trees (our outcome measure, labeled “VCA” throughout; out of a maximum possible 16,928 trees across all participants). On average, participants invested 82.63% of their 69€ endowment into the VCA, with 66.58% of participants choosing to invest their entire endowment into planting all possible 46 trees. Note that this extent of VCA is substantially higher than in previous studies, suggesting the VCA on offer in our study was unusually attractive to our participants. The average VCA does not differ by the participant’s gender (female participants: 37.79 trees vs. male: 38.47 trees; WMW, *p* = 0.724).

Following the literature (see Diederich and Goeschl 2014), we begin by examining which variables are predictive of VCA across conditions (see Appendix Table 13). Age is a significant predictor of VCA (coeff = 2.22, *p* = 0.042), whereas gender (coeff = 0.48, *p* = 0.757) and the participant’s number of kids are not significant (coeff = 0.92, *p* = 0.207). These results are all consistent with several past findings (Löschel et al. 2013; Diederich and Goeschl 2014 but see also Andre et al. 2021). A high school diploma (“High School Dipl.”) is associated with higher VCA (coeff = 10.68, *p* < 0.001), in line with Diederich and Goeschl (2014). Employment is also positively associated (coeff = 11.51, *p* = 0.001), as one might expect that being employed implies greater disposable income (Löschel et al. 2013). Meanwhile, neither risk nor patience preferences are significantly associated with VCA (Risk: coeff = 0.17, *p* = 0.553; Patience: coeff = − 0.22, *p* = 0.381). Living in a rural area or not does also have no significant effect (coeff = − 1.37, *p* = 0.328). Lastly, we find some variation by study location, which we discuss in more detail in Appendix A.

### Treatment Effects

**Fig. 1:**
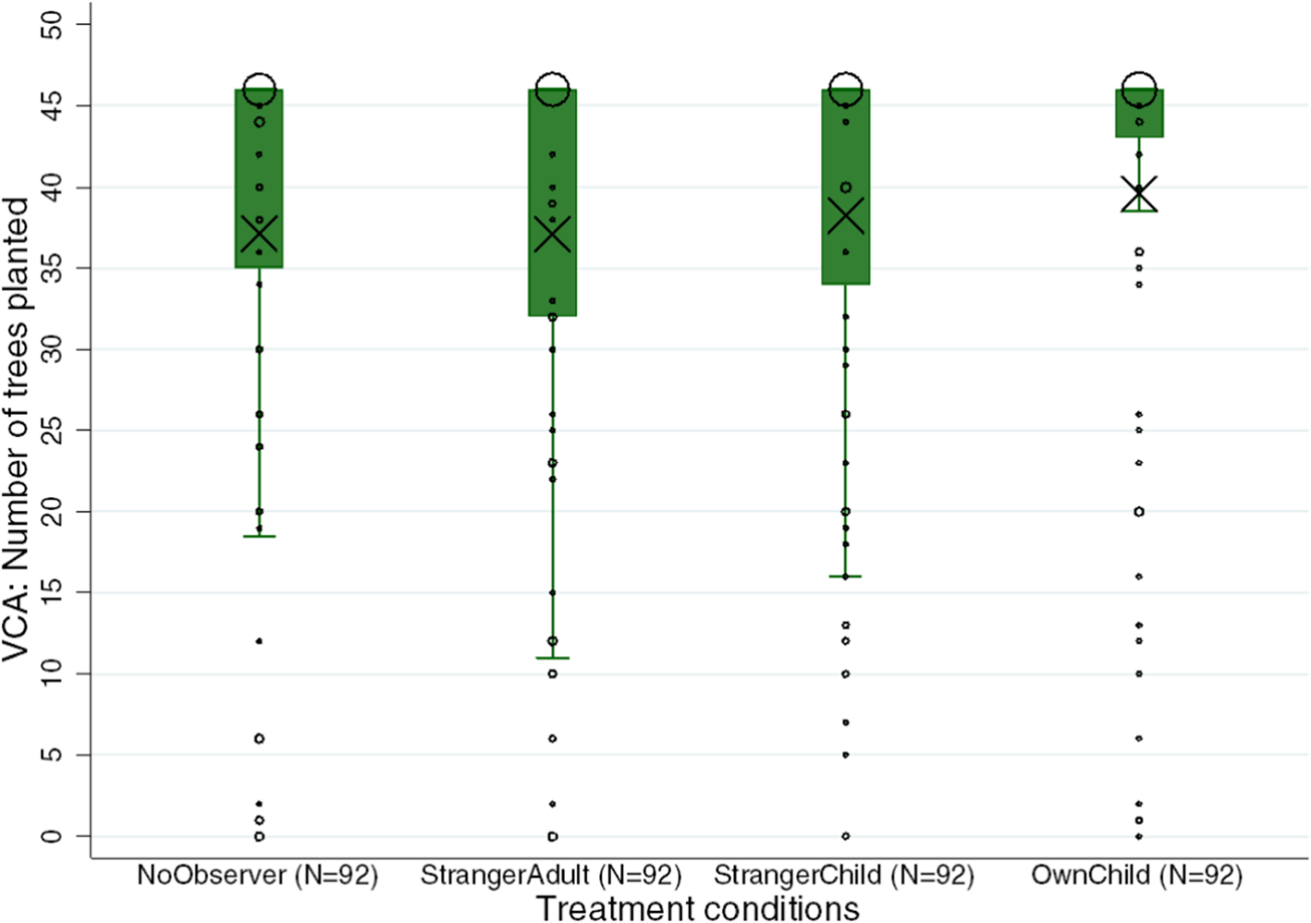
VCA: Number of trees planted by treatment condition (*N* = 368 subjects). Each box plot shows the average VCA of participants in each treatment. Box plots show the mean (indicated by black X signs), the 25th and 75th percentiles, Tukey whiskers (median ± 1.5 times the interquartile range), and individual data points. Larger dots indicate a higher number of participants who invested in the corresponding number of trees

Turning to our conditions, we first summarize the raw VCA values (see Fig. 1). We observe the lowest VCA in *NoObserver* (mean = 37.12, 25th percentile = 35.00 and 75th percentile = 46.00) and *StrangerAdult* (mean = 37.09, 25th percentile = 32.00, 75th percentile = 46.00). VCA is slightly higher in *StrangerChild* (mean = 38.24, 25th percentile = 34.00, 75th percentile = 46.00) and it is highest in *OwnChild* (mean = 39.60, 25th percentile = 43.00, 75th percentile = 46.00).

We next examine the effect of our treatments econometrically. As Table 2 shows, the largest coefficient relative to the baseline *NoObserver* is the *OwnChild* treatment. Without control variables, the *OwnChild* coefficient is positive but not significant (OLS: coeff = 2.48, *p* = 0.236; Tobit: coeff = 9.44, *p* = 0.149), whereas with control variables, the *OwnChild* treatment leads to significantly larger VCA (OLS: coeff = 3.69, *p* = 0.064; Tobit: coeff = 11.88, *p* = 0.050). Neither the coefficient on *StrangerChild* nor *StrangerAdult* is significant with or without control variables. Thus, in line with our descriptive and graphical results (Fig. 1), our econometric results (sometimes significantly but always directionally) suggest that *OwnChild* leads to the highest VCA, relative to the *NoObserver* baseline condition.

**Table 2:** Regression results for the entire sample

	(1)	(2)	(3)	(4)
	VCA	VCA	VCA	VCA
*OwnChild*	2.48 (2.09)	3.69* (1.98)	9.44 (6.52)	11.88** (6.03)
*StrangerChild*	1.12 (2.09)	2.05 (1.97)	4.16 (6.37)	5.75 (5.81)
*StrangerAdult*	− 0.03 (2.09)	2.53 (2.00)	2.02 (6.35)	9.61 (6.00)
*Age*		0.20* (0.11)		0.45 (0.33)
*Female*		0.68 (1.55)		3.67 (4.70)
*Nr. kids*		1.11 (0.73)		3.16 (2.20)
*Risk*		0.20 (0.29)		1.23 (0.87)
*Patience*		− 0.17 (0.25)		− 0.84 (0.76)
*High school dipl*		10.02*** (2.07)		22.78*** (5.67)
*Employed*		11.82*** (3.43)		25.63*** (9.21)
*Rural*		− 0.38 (1.44)		1.45 (4.31)
Constant	37.12*** (1.47)	6.03 (6.50)	57.12*** (4.81)	− 15.66 (19.05)
var(e.vca)			1315.15*** (209.16)	1008.47*** (159.22)
*N*	368	362	368	362
Location fixed effects	No	Yes	No	Yes

Ordinary least squares ((1)–(2)) and tobit regressions ((3)–(4)); upper limit 46 and lower limit 0). *p < 0.10, **p < 0.05, ***p < 0.01. The columns with covariates have slightly reduced sample size since, due to a technical glitch, the educational attainment level was not recorded for five participants, and one participant did not complete the full questionnaire. *OwnChild*, *StrangerChild*, and *StrangerAdult* equals 1 for the respective treatment and 0 otherwise (baseline is the *NoObserver* treatment). Age is measured in years. Female equals 1 for female participants. The number of kids controls for the respective variable for each participant. Risk measures self-assessed risk attitudes with higher values indicating higher risk-seeking. Patience measures self-assessed time preferences with higher values indicating higher patience. High School Dipl. is equal to 1 for participants who completed secondary education and 0 otherwise. Employed is equal to 1 if a participant is employed and 0 otherwise. Rural is equal to 1 for participants living in rural areas and 0 for those living in a city. Location Fixed Effects include a categorical variable controlling for the study locations Rathausgalerien, Herbstmesse, and Sillpark

These results suggest directionally that parents may be affected by their own children’s presence when making the VCA decision, but not with other observers present. To isolate the potential mechanisms at work, we explore three explanations using pre-registered non-parametric tests. First, we investigate to what extent the genetic link in particular matters, holding constant the “observer’s generation”. While VCA is higher, as predicted, in *OwnChild* (39.60 trees planted) than in *StrangerChild* (38.24 trees planted), this difference is not significant (WMW, *p* = 0.419).

Second, we test whether a representative of the future generation as an observer has a larger impact on VCA than an adult observer. We pool VCA across the two treatments, in which a child is the observer (*OwnChild* and *StrangerChild*) and compare it with VCA in *StrangerAdult*. Again, as expected, the average VCA is higher (38.92 trees planted) when being observed by a child, but not significantly different from the average VCA (37.09 trees planted) when being observed by an adult (WMW, *p* = 0.471).

Finally, we investigate a general observability effect, comparing VCA in *NoObserver* with the average VCA from across the three treatments with observers (*OwnChild*, *StrangerChild*, and *StrangerAdult*). Even though VCA is higher, the difference between the pooled observer conditions (38.31 trees planted) and the *NoObserver* condition (37.12 trees planted) is also not significant (WMW, *p* = 0.328).

### Treatment Effects by Education

**Fig. 2:**
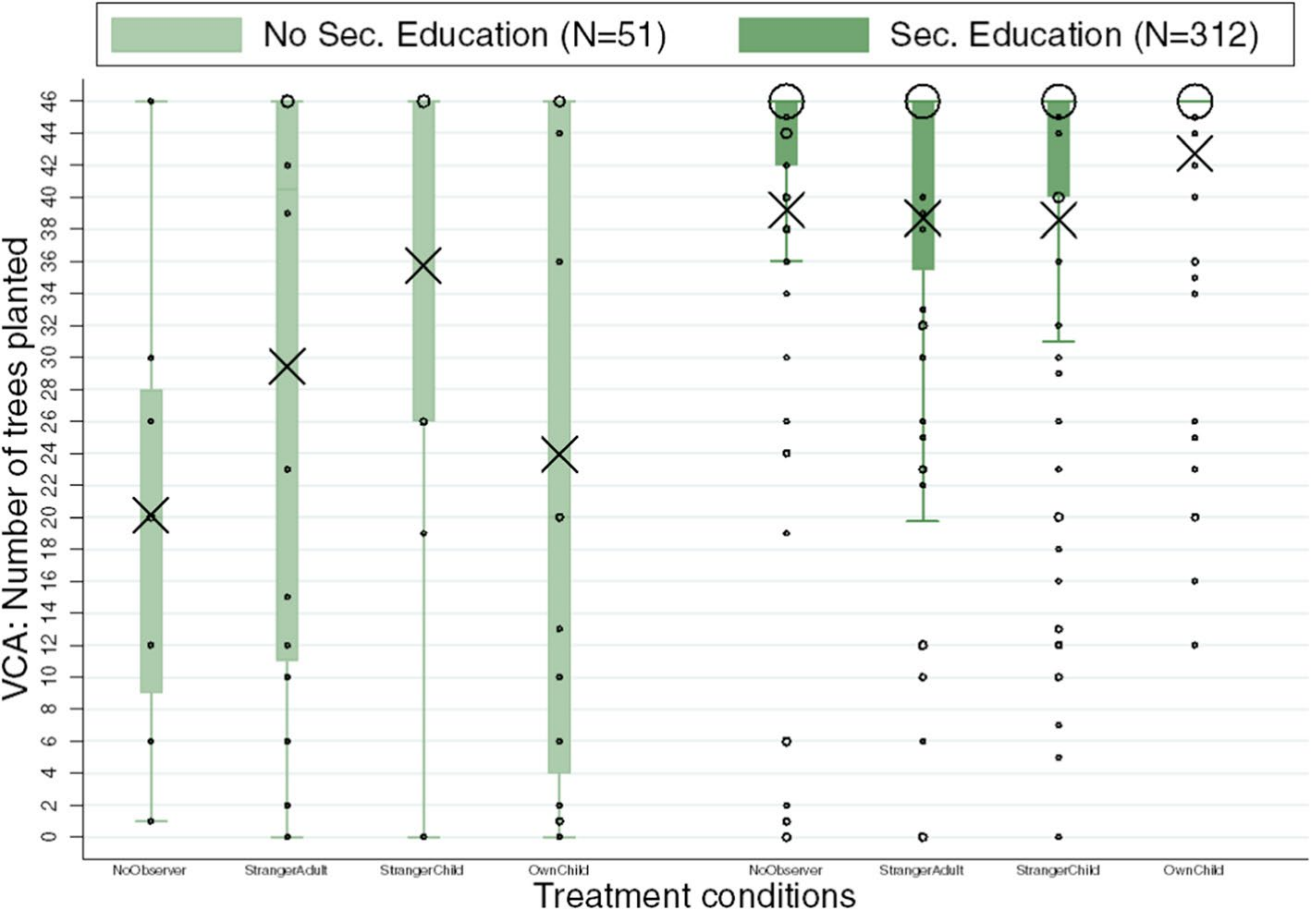
VCA: Number of trees planted, by condition and education (*N* = 363 subjects). Each set of four box plots shows the average VCA of participants for each education level. In each education subplot, the order of conditions is as follows: *NoObserver*, *StrangerAdult*, *StrangerChild*, and *OwnChild*. Box plots show the mean (indicated by black X signs), the 25th and 75th percentiles, Tukey whiskers (median ± 1.5 times the interquartile range), and individual data points. Larger dots indicate a higher number of participants with the corresponding number of trees

We found education to be an important determinant of the willingness to invest in VCA, in line with prior work (Diederich and Goeschl 2014). Thus, we examine our treatment effects in two sub-analyses for participants with versus without high school diploma (see Appendix Table 14 for descriptive statistics by educational background). Average VCA by treatment and high school diploma groups are graphically summarized in Fig. 2. First, we observe a substantial main effect of having a high school diploma, pooled across treatments, consistent with prior research (Diederich and Goeschl 2014): whereas participants with secondary education invested in planting 39.37 trees on average (25th percentile = 40.00, 75th percentile = 46.00), participants without secondary education invested at a significantly lower rate of 27.61 trees (25th percentile = 10.00, 75th percentile = 46.00; WMW, *p* < 0.001).

Participants with a high school diploma constitute the majority of our sample (312 of 368 participants, or 86%). Focusing on these participants first, we observe consistent and sizeable effects of the *OwnChild* treatment: across all specifications, parents who are observed by their own child are significantly more likely to invest in VCA (see *OwnChild* coefficient all columns in Table 3). We do not find any evidence that being observed by a stranger adult or stranger child leads to higher VCA. Consistent with our results across the entire sample, these findings suggest that the *OwnChild* condition leads to the highest VCA for more educated parents.

**Table 3:** Regression results for parents with a high school diploma

	(1)	(2)	(3)	(4)
	VCA	VCA	VCA	VCA
*OwnChild*	3.52* (2.01)	5.17*** (1.98)	13.73* (7.25)	18.14** (7.07)
*StrangerChild*	− 0.62 (1.95)	0.73 (1.92)	− 0.83 (6.50)	3.50 (6.32)
*StrangerAdult*	− 0.51 (1.98)	2.10 (2.00)	1.01 (6.70)	11.23 (6.95)
*Age*		0.04 (0.11)		0.12 (0.36)
*Female*		0.52 (1.52)		2.94 (5.20)
*Nr. kids*		1.55** (0.74)		4.55* (2.60)
*Risk*		0.21 (0.29)		1.32 (0.99)
*Patience*		− 0.20 (0.24)		− 1.12 (0.85)
*Employed*		16.43*** (3.85)		38.64*** (11.79)
*Rural*		− 1.45 (1.45)		− 0.93 (4.95)
Constant	39.20*** (1.37)	18.92*** (6.54)	61.59*** (5.15)	8.07 (21.60)
var(e.vca)			1180.56*** (217.36)	1029.40*** (189.68)
*N*	312	311	312	311
Location fixed effects	No	Yes	No	Yes

Ordinary least squares ((1)–(2)) and tobit regressions ((3)–(4)); upper limit 46 and lower limit 0). *p < 0.10, **p < 0.05, ***p < 0.01. The columns with covariates have slightly reduced sample size since, among participants with a high school diploma, one participant did not complete the full questionnaire. *OwnChild*, *StrangerChild*, and *StrangerAdult* equals 1 for the respective treatment and 0 otherwise (baseline is the *NoObserver* treatment). Age is measured in years. Female equals 1 for female participants. The number of kids controls for the respective variable for each participant. Risk measures self-assessed risk attitudes with higher values indicating higher risk-seeking. Patience measures self-assessed time preferences with higher values indicating higher patience. Employed is equal to 1 if a participant is employed and 0 otherwise. Rural is equal to 1 for participants living in rural areas and 0 for those living in a city. Location Fixed Effects include a categorical variable controlling for the study locations Rathausgalerien, Herbstmesse, and Sillpark

In fact, we also find evidence that parents invest uniquely more when being observed by their own child than being observed by a stranger child (WMW, *p* = 0.031). This result supports that, for more educated parents, the genetic link between a parent and their own child uniquely matters for VCA, even when holding constant that the observer is a representative of a future generation.

Turning to participants without a high school diploma (*N* = 51), we find that the treatment effects look qualitatively different. Specifically, the average VCA is low in the *NoObserver* condition (20.13) and, remarkably, also in the *OwnChild* condition (23.94). The highest mean VCA is observed in the *StrangerChild* condition (35.73), which is significantly different from the *NoObserver* condition without covariates and with covariates using Tobit (see columns 1, 3, and 4 in Table 4) but not significant with covariates using OLS (in column 2, *p* = 0.104). The *StrangerAdult* condition (29.44) falls in the middle.

**Table 4:** Regression results for parents without a high school diploma

	(1)	(2)	(3)	(4)
	VCA	VCA	VCA	VCA
*OwnChild*	3.81 (7.72)	0.20 (8.33)	6.30 (12.58)	0.17 (12.12)
*StrangerChild*	15.60* (8.28)	14.49 (8.69)	27.94* (14.37)	22.70* (13.20)
*StrangerAdult*	9.31 (7.72)	2.96 (7.74)	15.12 (12.74)	3.40 (11.27)
*Age*		1.20*** (0.41)		2.07*** (0.69)
*Female*		4.94 (6.16)		10.93 (9.83)
*Nr. kids*		0.26 (2.49)		− 0.38 (3.75)
*Risk*		1.20 (1.01)		2.21 (1.63)
*Patience*		− 1.78 (1.12)		− 2.76 (1.71)
*Employed*		3.30 (8.98)		6.46 (13.09)
*Rural*		5.08 (6.20)		8.02 (9.34)
Constant	20.13*** (6.30)	− 32.63 (20.81)	22.10** (10.15)	− 71.33** (33.20)
var(e.vca)			800.58*** (241.69)	541.55*** (161.35)
N	51	51	51	51
Location fixed effects	No	Yes	No	Yes

Ordinary least squares ((1)–(2)) and tobit regressions ((3)–(4)); upper limit 46 and lower limit 0). Standard errors in parentheses. *p < 0.10, **p < 0.05, ***p < 0.01. *OwnChild*, *StrangerChild*, and *StrangerAdult* equals 1 for the respective treatment and 0 otherwise (baseline is the *NoObserver* treatment). Age is measured in years. Female equals 1 for female participants. The number of kids controls for the respective variable for each participant. Risk measures self-assessed risk attitudes with higher values indicating higher risk-seeking. Patience measures self-assessed time preferences with higher values indicating higher patience. Rural is equal to 1 for participants living in rural areas and 0 for those living in a city. Location Fixed Effects include a categorical variable controlling for the study locations Rathausgalerien, Herbstmesse, and Sillpark

### Treatment Effects by Climate Change Perception

Following the experimental intervention and VCA task, participants completed a short survey which included a questionnaire based on Howe et al. (2015). In this questionnaire, participants were asked about their attitudes towards climate change, allowing us to test how our treatment effects vary by differing climate change perceptions. To reduce variance from any single question item, we constructed an index—which we refer to as climate change perception index—based on the following three questions from Howe et al. (2015): First, “What do you think: is global warming happening?” with three possible answers: “Yes”, “No” and “I don’t know”. Second, “Which of the following statements do you most agree with? Global warming …” with answers: “…is mainly caused by human activities.”, “…is mainly caused by human activities and natural changes.”, “…is mainly caused by natural changes in the environment.“, and”…does not take place.“. Third, “Which statement is closest to your opinion?” with answers: “Most scientists believe that global warming takes place.”, “There is a lot of disagreement among scientists about whether global warming occurs.”, “Most scientists do not believe that global warming takes place.” and “I don’t know.”

The higher the climate change perception index, the more the participant is convinced by climate change and the human contribution to it: specifically, if a participant chose “Yes” to question 1, “…is mainly caused by human activities.” to question 2 and “Most scientists believe that global warming takes place.” to question 3, the index is equal to 3. If they chose two out of these three answers, the index is equal to 2. If they chose only one out of the three, the index is equal to 1; and 0 if none of those answers was chosen. Based on this climate change perception index, 41% of all participants show some skepticism towards climate change (i.e. received an index lower than 3). We observe that VCA is significantly lower for those classified as skeptical (climate change perception index < 3) than those who believe in climate change (climate change perception index = 3) (35.68 vs. 39.56, respectively; WMW, *p* = 0.023).

We conducted subgroup analyses for those who received an index equal to 3 (i.e. convinced that climate change is happening, mainly caused by humans and that most scientists agree) versus lower than 3 (i.e. skeptical of climate change). As Table 5 shows, interesting but somewhat opposing findings emerge: participants with a climate change perception index below 3 are not more likely to give when their own child is observing them, but perhaps more so when a genetically unrelated child or another adult is watching them. In contrast, participants with a climate change perception index of 3 are only affected by the presence of their own child but not affected by other children or adults. All these effects are directionally the same but smaller in magnitude and less likely to be significant without control variables (columns 1 and 3) but become statistically significant when fixed effects and control variables are included in the regressions (columns 2 and 4).

**Table 5:** Regression results depending on the climate index

	(1)	(2)	(3)	(4)
	Climate change perception index < 3		Climate change perception index = 3	
	VCA	VCA	VCA	VCA
*OwnChild*	9.22 (9.56)	12.76 (8.96)	14.62 (9.03)	18.03** (8.53)
*StrangerChild*	18.58* (10.33)	19.22** (9.58)	− 6.20 (7.69)	− 2.36 (7.10)
*StrangerAdult*	8.86 (10.71)	18.58* (10.26)	− 4.21 (7.51)	5.87 (7.23)
*Age*		0.06 (0.54)		0.47 (0.40)
*Female*		1.06 (7.20)		3.68 (6.02)
*Nr. kids*		0.27 (2.98)		8.58** (3.42)
*Risk*		− 0.33 (1.40)		2.60** (1.07)
*Patience*		− 0.45 (1.23)		− 1.39 (0.98)
*High school dipl*		23.95*** (7.97)		17.09** (8.62)
*Employed*		27.33** (13.41)		32.19** (13.38)
*Rural*		− 0.30 (6.77)		1.54 (5.34)
Constant	46.31*** (7.22)	1.35 (29.55)	63.61*** (6.18)	− 29.85 (24.53)
var(e.vca)	1424.04*** (335.01)	1110.84*** (259.24)	1097.97*** (236.00)	783.12*** (166.83)
N	150	150	218	212
Location fixed effects	No	Yes	No	Yes

Tobit regressions for those with a climate change perception index lower 3 (see columns (1)–(2)) and equal to 3 (see columns (3)–(4)); upper limit 46 and lower limit 0. Standard errors in parentheses. *p < 0.10, **p < 0.05, ***p < 0.01. *OwnChild*, *StrangerChild*, and *StrangerAdult* equals 1 for the respective treatment and 0 otherwise (baseline is the *NoObserver* treatment). Age is measured in years. Female equals 1 for female participants. The number of kids controls for the respective variable for each participant. Risk measures self-assessed risk attitudes with higher values indicating higher risk-seeking. Patience measures self-assessed time preferences with higher values indicating higher patience. Rural is equal to 1 for participants living in rural areas and 0 for those living in a city. Location Fixed Effects include a categorical variable controlling for the study locations Rathausgalerien, Herbstmesse, and Sillpark

Interestingly, participants with a climate change perception index below 3 show a similar pattern to those without a high-school diploma, while participants with a climate change perception index of 3 behave similarly to participants with a high-school diploma. Indeed, we observe a positive correlation between higher education and the climate index (Spearman's rho = 0.233, *p* < 0.001), which is also consistent with past literature (Stevenson et al. 2014; Lee et al. 2015).^1^

## Discussion

In an intergenerational public good (for example, planting trees that offset CO2 emissions), the beneficiaries (future generations) are not the same as the decision-makers (current generation). Parents, who have a genetic link to the future through their children, would be particularly likely to invest in VCA. Indeed, we find a high willingness to invest in VCA, with over 80% of all parents investing in the VCA to plant trees. This is more than the usual VCA contributions found in the literature: Bruns et al. (2018) report that participants spent 35% of a default amount of money on VCA, while Diederich and Goeschl (2014) find that only 16% of subjects chose the emission reduction instead of a cash amount. It is possible that our participants were more willing to invest in VCA because we designed the VCA based on some of the “best practices” from the VCA literature (see the Methods section). However, in addition to offsetting CO2 emissions, it is possible that planting more trees in the surrounding area of Innsbruck (where the experiment took place) may also provide recreational value to many of our participants (see, e.g., Pittel and Rübbelke 2008; Baranzini et al. 2018). Or that planting trees may be a form of CO2 reducing activity that is perceived as more transparent, trustworthy, and less abstract than investments in other technologies to reduce CO2 emissions (Schwirplies et al. 2019). Our findings, in combination with the literature, suggest that there are already many lessons to be learned from the prior literature to make VCAs attractive.

We proposed that VCA would be heightened when a parent is being observed by their own offspring. The parent’s own child would serve multiple purposes, most importantly as a reminder of the fact that a (genetic) link connects the parent (decision-maker) to their own child (future beneficiary). Across our entire sample, we find some evidence that parents give more when their children are watching their VCA decision but, importantly, education plays a key role: as Diederich and Goeschl (2014) note, participants with a high school diploma usually exhibit greater willingness to engage in VCA, and, in our setting, our treatment effects are substantially larger in the subsample of participants with a high school diploma. While it is unclear why educated parents respond more to the treatment, it may be that their educational background means that they are more aware of the environmental impact of their decision (as reflected in their higher climate change perception index) or because better education is usually correlated with more disposable income, which means that sacrificing the experimental endowment as an investment into the future would affect their finances today less than those of less-educated (and potentially lower-earning) parents. Future research is needed to fully explore these effects.

Similarly, our study points to the importance of beliefs and perceptions about climate change in understanding the treatment effects. Using questions first used in a well-known study by Howe et al. (2015), we show that those who believe climate change is happening, is mostly caused by human activities and that scientists are in agreement that climate change is particularly affected by their own child’s presence when deciding to invest in VCA. On the other hand, those who are skeptical of climate change are, surprisingly, mostly affected by other children (to whom they are not related) or other adults. This suggests that social pressure to invest into climate-mitigating strategies may be better applied to this skeptical group by members, not of their “in-group” or close family. It is possible that climate change-skeptical parents think that their children agree with them, would judge them less harshly for giving to this cause, or believe that they could convince them of their merits of such a decision, which they may not be able to do to unrelated observers.

Our study makes several contributions to the literature. First, we contribute to the literature on VCA. Previous studies have investigated both the personal characteristics that determined engagement with VCA (Diederich and Goeschl 2014) and contextual cues—often in the form of nudges—that can lead to more VCA (Araña and León 2013; Böhm et al. 2020; Carattini and Blasch 2020). In this paper, we focus instead on a novel context that we use as an intervention—the role of the genetic link across generations. Since VCAs are intergenerational by nature, we argue that VCA interventions can benefit from considering the intergenerational structure of families and we demonstrate that parents are indeed more willing to invest in future public goods when they are observed by their children, not just other adults or children.

Second, we contribute to the burgeoning literature on intergenerational public goods. Previous research has studied resource replenishment rates (Fischer et al. 2004), institutions (Hauser et al. 2014), and peer punishment (Lohse and Waichman 2020). However, a previously neglected aspect of intergenerational public goods is relatedness (Nowak 2006): decision-makers may not be present to reap the benefits of their actions in the future, but their own descendants could benefit. As a result, genetic offspring should be considered in other interventions to increase contributions to intergenerational public goods.

Furthermore, our study also speaks to the standard public goods game: although observability is a widely studied intervention in economics (Hoffman et al. 1996), our study suggests that the type of observers matters: while adults are typically recruited for studies using observability, we show that variation in observers can yield differing results. In our setting, adult observers did not affect VCA, either in the main analysis or the subgroup analyses. Our findings document the importance of choosing an observer that manipulates the theoretical construct in question.

Finally, we contribute to the literature on child-parent interactions. Most work has investigated one direction of this causal relationship—how parents influence their children—such as, for example, the extent to which parents’ sharing behavior in the dictator game influences their child’s subsequent dictator game behavior (Ben-Ner et al. 2017). Similarly, prior fieldwork has found that preferences are shaped by one’s parents’ behavior in childhood and persist into adulthood (Fernández et al. 2004). Here, we reverse the causal direction of this relationship, finding that parents’ behavior can be shaped by their children. Our intervention is relatively minimal and only involves observation by children, leaving open the possibility that children’s actual influence on their parents is much larger in reality.

Our findings have implications for policy-makers and offer new research questions for scholars across a variety of domains. We focused on VCA, specifically planting trees, for which we found a notably high willingness to invest into the future. Based on our fixed price for reducing one ton of CO2 (by design, fixed at 100€ per ton) and the average CO2 reduction for each tree (0.015 tons per year), we can estimate that our participants were willing to offset 0.570 tons of CO2 annually.^2^ Extrapolating these annual reductions into the future, our participants, on average, invested into offsetting 68.42 tons of CO2 over the expected lifetime of these trees of about 120 years. We believe these estimates represent meaningful ecological and economic trade-offs, which should afford policy-makers some hope that citizens are willing to incur costs today to help the future. Indeed, Steinke and Trautmann (2021) find equally high willingness among their respondents who are willing to incur a cost today to help the future and even the far future (from which they themselves and even their own kin no longer benefit from).

However, parents make many more important decisions in daily life that have consequences, if not always for future generations, at least for years and decades to come that also shape the lives of the next generation. Consider, for instance, voting: in many countries, adults are not allowed to take their children into the voting booth. Would parties that emphasize long-term investments in education and environmental protection receive a greater voting share if parents had to choose under the watchful eyes of their own children? While this is an open empirical question, one could imagine that voting systems may take such considerations into account (Kamijo et al. 2017). Ultimately, this perspective can be extended further, including to seemingly mundane activities, such as shopping for groceries (e.g., buying meat or vegetarian alternatives), or choosing whether to take the bike to work or on the school run: children may be a powerful, yet underappreciated way to shape their parent’s behavior.

## Figures and Tables

**Fig. 3:**
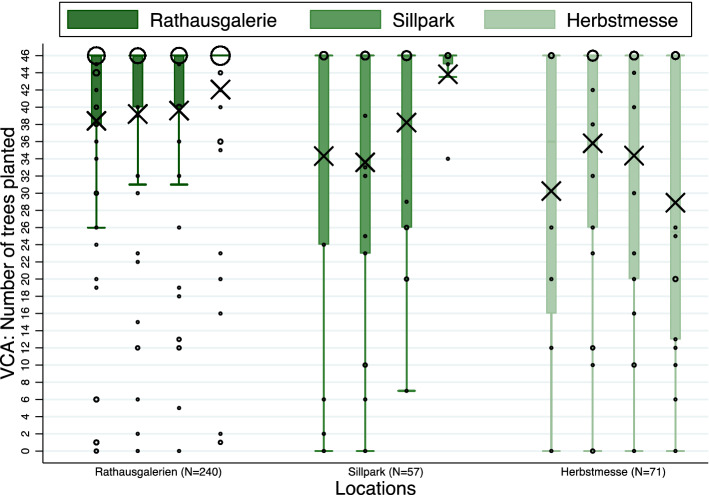
VCA: Number of trees planted by location and treatment condition (N = 368 subjects). Each set of four box plots shows the average VCA of participants in each location. Separate treatment order for each location: NoObserver, StrangerAdult, StrangerChild, and OwnChild. Box plots show the mean (indicated by black X signs), the 25th and 75th percentiles, Tukey whiskers (median ± 1.5 times the interquartile range), and individual data points. Larger dots indicate a higher number of participants with the corresponding number of trees

**Fig. 4:**
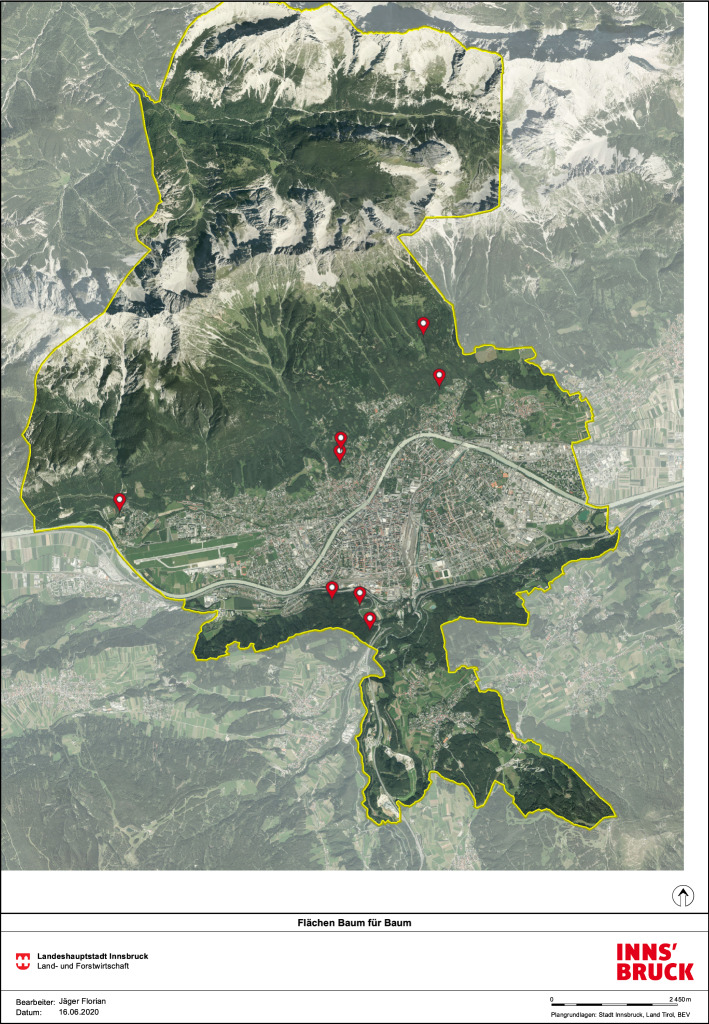
Area for the project’s foresting program. *Source*: WDB Walddatenbank WebGIS

**Fig. 5:**
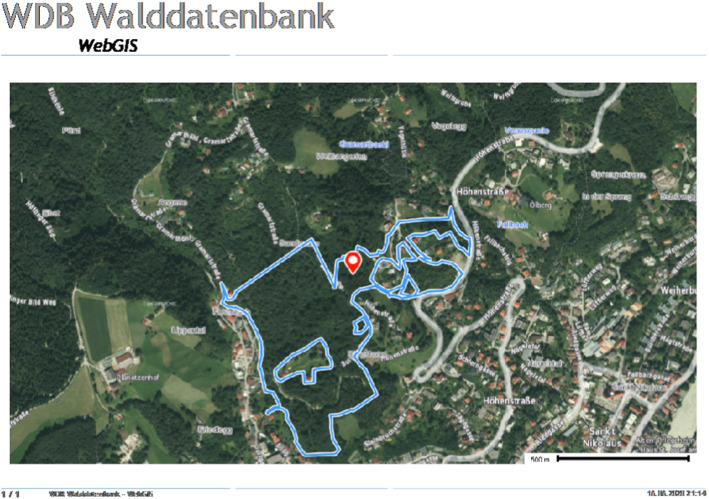
Area of the StadtKlimaWald. *Source*: WDB Walddatenbank WebGIS

## Data Availability

All data and documentation used in the analysis will be shared after publication at https://osf.io/2kdgz/?view_only=118e3af382284948835e8e66b4d1f451.

## Appendix A: Additional Analyses by Study Location

In addition to the subgroup analyses by the educational background in the main text, we analyze our treatment effects by study location. See Table 6 for the observations by location and treatment.

**Table 6:** Observations by location and treatment

Treatments	Rathausgalerien	Herbstmesse	Sillpark	Total
*NoObserver*	71	8	13	92
*StrangerAdult*	47	26	19	92
*StrangerChild*	54	19	19	92
*OwnChild*	68	18	6	92
	240	71	57	368

The table presents amounts of observations by treatment and location. These amounts significantly differ across treatments and locations (chi2 *p* = *0.001*)

Investigating the average *VCA* across locations (see Fig. 3), we find that VCA differs significantly by location (kwallis, *p* = *0.004*). On average, VCA is highest at the Rathausgalerien (39.87 trees planted), followed by Sillpark (36.37) and Herbstmesse (33.04). Figure 3 also shows that two locations (Rathausgalerien and Sillpark) look qualitatively similar to each other and generally in line with our anticipated treatment effects (with the *OwnChild* condition showing the largest effect), while Herbstmesse looks qualitatively different (in particular the *OwnChild* condition). To shed more light on this heterogeneity, we repeat our main analysis for each study location separately, using the same regression specifications as in the main paper (see Tables 4, 5 and 6).

We begin with the Rathausgalerien location, which is our largest study location, where 65.22% of all participants (*N* = 240) were recruited. As Table 7 shows, across all specifications, we find that the coefficient for *OwnChild* is positive and significant. Without control variables, the effect is significant at the 10% level using OLS (coeff = 3.65, *p* = 0.094) and at the 5% level using Tobit (coeff = 16.34, *p* = 0.032). Once we include control variables, the coefficient on *OwnChild* is significant at the 1% level for both OLS (coeff = 5.46, *p* = 0.007) and Tobit (coeff = 19.82, *p* = 0.004). These results suggest that *OwnChild* has a positive and significant effect on VCA. Indeed, comparing the *OwnChild* coefficient with the *StrangerChild* coefficient suggests that this effect is uniquely driven by observation from the parent’s own child (*p* = 0.085), ruling out the explanation that the parent participant acts when any child who is a representative of the future generation is present.

Turning to the second (Sillpark) and third (Herbstmesse) location, we find no significant effects for any treatments in either location (see Tables 8 and 9, respectively). In the case of the Sillpark location, the coefficients for *OwnChild* and *StrangerChild* are positive and generally similar to the Rathausgalerien location but not significant. However, the coefficients in the Herbstmesse location are notably different (and, in the *OwnChild* condition, even negative but not significantly so) from the other two locations.

**Table 7:** Regression results for location Rathausgalerien

	(1)	(2)	(3)	(4)
	VCA	VCA	VCA	VCA
*OwnChild*	3.65* (2.17)	5.46*** (2.02)	16.34** (7.59)	19.82*** (6.76)
*StrangerChild*	1.20 (2.31)	1.93 (2.13)	6.09 (7.59)	7.34 (6.66)
*StrangerAdult*	0.80 (2.41)	2.87 (2.25)	7.71 (8.06)	14.44* (7.40)
*Age*		0.19 (0.13)		0.47 (0.41)
*Female*		2.90* (1.66)		7.73 (5.33)
*Nr. kids*		1.34 (1.01)		4.09 (3.33)
*Risk*		0.31 (0.32)		1.62 (1.06)
*Patience*		− 0.41 (0.27)		− 1.57* (0.93)
*High school dipl*		14.57*** (2.75)		34.25*** (7.80)
*Employed*		12.40*** (3.87)		26.39** (10.81)
*Rural*		− 0.52 (1.54)		1.87 (5.01)
Constant	38.41*** (1.52)	0.29 (7.44)	58.11*** (5.36)	− 36.72 (23.02)
var(e.vca)			1191.66*** (247.49)	827.32*** (168.78)
N	240	237	240	237

Ordinary least squares ((1)–(2)) and tobit regressions ((3)–(4)); upper limit 46 and lower limit 0). **p* < 0.10, ***p* < 0.05, ****p* < 0.01. *StrangerAdult*, *StrangerChild*, *OwnChild* equals 1 for the respective treatment and 0 otherwise (baseline is the *NoObserver* treatment). Age is measured in years. Female equals 1 for female participants. The number of kids controls for the respective variable for each participant. Risk measures self-assessed risk attitudes with higher values indicating higher risk-seeking. Patience measures self-assessed time preferences with higher values indicating higher patience. High School Dipl. is equal to 1 for participants who completed secondary education and 0 otherwise. Employed is equal to 1 if a participant is employed and 0 otherwise. Rural is equal to 1 for participants living in rural areas and 0 for those living in a city

**Table 8:** Regression results for location Sillpark

	(1)	(2)	(3)	(4)
	VCA	VCA	VCA	VCA
*OwnChild*	9.53 (7.44)	9.27 (7.61)	14.50 (21.60)	18.78 (21.78)
*StrangerChild*	3.90 (5.43)	4.51 (5.84)	6.96 (15.44)	9.79 (15.66)
*StrangerAdult*	− 0.73 (5.43)	1.47 (5.71)	− 7.26 (15.04)	− 1.01 (14.64)
*Age*		0.36 (0.27)		1.70* (0.98)
*Female*		− 3.10 (5.13)		1.90 (14.43)
*Nr. kids*		− 1.27 (1.56)		− 3.68 (4.05)
*Risk*		0.02 (0.84)		2.39 (2.49)
*Patience*		0.00 (0.76)		− 0.74 (2.02)
*High school dipl*		3.07 (5.05)		− 2.09 (13.29)
*Employed*		38.06** (16.09)		204.80 (5895.15)
*Rural*		− 1.28 (4.19)		− 7.71 (11.18)
Constant	34.31*** (4.18)	− 15.51 (22.27)	55.29*** (12.80)	− 216.75 (5895.40)
var(e.vca)			1263.25** (488.91)	1034.57** (409.92)
N	57	56	57	56

Ordinary least squares ((1)–(2)) and tobit regressions ((3)–(4)); upper limit 46 and lower limit 0). **p* < 0.10, ***p* < 0.05, ****p* < 0.01. *StrangerAdult*, *StrangerChild*, *OwnChild* equals 1 for the respective treatment and 0 otherwise (baseline is the *NoObserver* treatment). Age is measured in years. Female equals 1 for female participants. The number of kids controls for the respective variable for each participant. Risk measures self-assessed risk attitudes with higher values indicating higher risk-seeking. Patience measures self-assessed time preferences with higher values indicating higher patience. High School Dipl. is equal to 1 for participants who completed secondary education and 0 otherwise. Employed is equal to 1 if a participant is employed and 0 otherwise. Rural is equal to 1 for participants living in rural areas and 0 for those living in a city

**Table 9:** Regression results for Herbstmesse location

	(1)	(2)	(3)	(4)
	VCA	VCA	VCA	VCA
*OwnChild*	− 1.36 (6.98)	− 9.14 (7.59)	− 1.93 (16.43)	− 18.11 (16.24)
*StrangerChild*	4.12 (6.93)	2.03 (7.08)	7.54 (16.47)	− 0.55 (15.59)
*StrangerAdult*	5.56 (6.65)	2.61 (6.73)	12.43 (15.97)	4.27 (14.90)
*Age*		− 0.13 (0.32)		− 0.60 (0.73)
*Female*		− 4.19 (4.67)		− 5.91 (10.57)
*Nr. kids*		4.68** (1.96)		10.29** (4.77)
*Risk*		0.66 (0.89)		1.68 (1.98)
*Patience*		0.15 (0.70)		0.09 (1.56)
*High school dipl*		11.45** (4.68)		25.55** (10.55)
*Employed*		8.29 (7.78)		14.11 (16.73)
*Rural*		4.06 (4.82)		10.11 (10.07)
Constant	30.25*** (5.81)	7.56 (17.24)	41.24*** (13.92)	4.90 (37.37)
var(e.vca)			1226.66*** (388.71)	852.68*** (266.53)
N	71	69	71	69

Ordinary least squares ((1)–(2)) and tobit regressions ((3)–(4)); upper limit 46 and lower limit 0). **p* < 0.10, ***p* < 0.05, ****p* < 0.01. *StrangerAdult*, *StrangerChild*, *OwnChild* equals 1 for the respective treatment and 0 otherwise (baseline is the *NoObserver* treatment). Age is measured in years. Female equals 1 for female participants. The number of kids controls for the respective variable for each participant. Risk measures self-assessed risk attitudes with higher values indicating higher risk-seeking. Patience measures self-assessed time preferences with higher values indicating higher patience. High School Dipl. is equal to 1 for participants who completed secondary education and 0 otherwise. Employed is equal to 1 if a participant is employed and 0 otherwise. Rural is equal to 1 for participants living in rural areas and 0 for those living in a city

While we cannot conclusively say what causes these differences in outcomes with respect to locations, we find significant differences across several participants’ demographics between locations. Specifically, significantly more participants recruited at Rathausgalerien have completed secondary education, live in an urban area, and they are slightly older than participants at Sillpark and Herbstmesse (see Table 12 for details). These participants may also be wealthier due to these characteristics (Psacharopoulos and Patrinos 2018; Rechnungshof Österreich 2020). This difference in demographics may in large part be driven by the physical location of the Rathausgalerien, which is inside a shopping mall near the mayor’s office in the city center of the state’s capital, Innsbruck. This shopping mall is frequented by people who live in the city and less so by people from out of town (for whom finding parking may be an issue in the center of town).

While both Sillpark and Herbstmesse are also located in the same city, the former is a shopping mall in a less central location, while the latter draws a substantially different crowd than the other two: our third study location “Herbstmesse” is also the name of an annual fair every fall that showcases household, home improvement, and gardening tools from local and international sellers. The fair draws people from all across the state, many of which travel from rural villages to the capital to visit this fair. The difference in observables in the Herbstmesse location is therefore not a surprise, nor are the smaller differences of the Sillpark location compared to the Rathausgalerien. In sum, these differences in observables may contribute to why there exists heterogeneity of treatment effects by treatment.

## Appendix B: Additional Tables

See Tables 10, 11, 12, 13, 14 and 15.

**Table 10:** Background information on participants

Variable	N	Total	Min	Max
% Female	368	67.39	0	1
Age	367	42.15 (6.808)	27	74
Number of kids	365	2.06 (0.971)	1	7
% Employed	362	95.58	0	1
% High school dipl	363	85.95	0	1
% University degree	363	42.98	0	1
% Married	362	66.30	0	1
Risk preference	365	5.350 (2.483)	0	10
Time preference	365	5.923 (2.846)	0	10
% Rural	362	50.55	0	1

The table presents mean values (with standard deviations in parentheses) or percentages. The number of observations varies slightly across questions due to some participants not filling out the complete questionnaire and not all questions being mandatory

**Table 11:** Background information on participants by treatment

Variable	N	*NoObserver*	N	*StrangerAdult*	N	*StrangerChild*	N	*OwnChild*	*p-*value
% Female	92	66.30	92	66.30	92	66.30	92	67.39	0.961
Age	91	42.89 (7.137)	92	41.67 (7.402)	92	41.62 (6.851)	92	42.42 (5.757)	0.450
Number of kids	92	2.076 (0.815)	92	1.956 (0.993)	92	2.065 (1.025)	89	2.157 (1.043)	0.431
% Employed	91	97.80	91	93.41	92	97.83	88	93.18	0.224
% High school dipl	91	91.21	92	82.61	92	88.04	88	81.82	0.206
% University degree	91	50.55	92	39.13	92	43.48	88	38.64	0.343
% Married	91	64.84	91	61.54	92	68.48	88	70.45	0.603
Risk preference	92	5.000 (2.660)	92	5.656 (2.508)	92	5.652 (2.241)	89	5.180 (2.488)	0.254
Time preference	92	5.565 (2.906)	92	6.228 (2.677)	92	6.109 (2.873)	89	5.786 (2.921)	0.397
% Rural	91	47.25	91	53.35	92	50.00	88	51.14	0.848

The table presents mean values (with standard deviations in parentheses) or percentages. *p* values testing equality across treatments, based on Fisher’s exact tests, except for variables age, the number of kids, risk attitudes, and time preferences (kwallis). The number of observations varies slightly across questions because some participants did not fill out the complete questionnaire, and not all questions being mandatory

**Table 12:** Background information on participants by location

Variable	N	Rathausgalerien	N	Herbstmesse	N	Sillpark	*p-*value
% Female	240	66.67	71	71.83	57	64.91	0.639
Age	239	42.76 (6.396)	71	40.65 (6.261)	57	41.30 (8.790)	0.018
Number of kids	238	1.975 (0.774)	70	2.128 (1.102)	57	2.351 (1.407)	0.547
% Employed	237	95.78	69	92.75	56	98.21	0.350
% High school dipl	237	91.56	69	75.36	57	75.44	0.000
% University degree	237	53.16	69	20.29	57	28.07	0.000
% Married	237	66.24	69	72.46	56	58.93	0.289
Risk preference	238	5.214 (2.448)	70	5.60 (2.312)	57	5.614 (2.814)	0.261
Time preference	238	5.702 (2.822)	70	6.414 (2.877)	57	6.246 (2.849)	0.086
% Rural	237	42.19	69	78.26	56	51.79	0.000

The table presents mean values (with standard deviations in parentheses) or percentages. *p* values testing equality across treatments, based on Fisher’s exact tests, except for variables age, the number of kids, risk attitudes, and time preferences (kwallis). The number of observations varies slightly across questions because some participants did not fill out the complete questionnaire, and not all questions being mandatory

**Table 13:** Correlational results for the entire sample (across all treatments)

	(1)	(2)	(3)	(4)
	VCA	VCA	VCA	VCA
*Age*	0.22** (0.11)	0.20* (0.11)	0.51 (0.34)	0.43 (0.33)
*Female*	0.48 (1.56)	0.64 (1.55)	2.67 (4.77)	3.33 (4.72)
*Nr. kids*	0.92 (0.73)	1.12 (0.73)	2.64 (2.21)	3.20 (2.19)
*Risk*	0.17 (0.29)	0.21 (0.29)	1.18 (0.88)	1.32 (0.87)
*Patience*	− 0.22 (0.25)	− 0.16 (0.25)	− 0.98 (0.78)	− 0.81 (0.77)
*High school dipl*	10.68*** (2.03)	9.69*** (2.06)	24.50*** (5.67)	21.70*** (5.64)
*Employed*	11.51*** (3.43)	11.40*** (3.42)	24.53*** (9.28)	24.35*** (9.18)
*Rural*	− 1.37 (1.40)	− 0.36 (1.44)	− 2.05 (4.22)	1.01 (4.32)
Constant	7.34 (6.28)	9.20 (6.27)	− 12.83 (18.75)	− 8.09 (18.50)
var(e.vca)			1060.21*** (167.94)	1025.28*** (162.04)
N	362	362	362	362
Location fixed effects	No	Yes	No	Yes

Ordinary least squares ((1)–(2)) and tobit regressions ((3)–(4)); upper limit 46 and lower limit 0). Standard errors in parentheses. **p* < 0.10, ***p* < 0.05, ****p* < 0.01. Age in years. Female equals 1 for female participants. The number of kids controls for the respective variable for each participant. Risk measures self-assessed risk attitudes with higher values indicating higher risk-seeking. Patience measures self-assessed time preferences with higher values indicating higher patience. High School Diploma. is equal to 1 for participants who completed secondary education and 0 otherwise. Employed is equal to 1 if a participant is employed and 0 otherwise. Location Fixed Effects include a categorical variable controlling for the study locations Rathausgalerien, Herbstmesse, and Sillpark. Rural is equal to 1 for participants living in rural areas and 0 for those living in a city

**Table 14:** Background information on participants by education

Variable	N	No high school dipl	N	High school dipl	*p*-value
% Female	51	70.59	312	66.99	0.747
Age	51	39.76 (6.860)	312	42.45 (6.635)	0.015
Number of kids	51	2.157 (1.084)	312	2.052 (0.954)	0.634
% Employed	51	90.20	311	96.46	0.059
% University degree	51	0 (0)	312	50.00	0.000
% Married	51	56.86	311	67.85	0.150
Risk preference	51	5.196 (2.646)	312	5.402 (2.438)	0.594
Time preference	51	6.333 (2.673)	312	5.862 (2.856)	0.302
% Rural	51	56.86	311	49.52	0.367

The table presents mean values (with standard deviations in parentheses) or percentages. *p* values testing equality across treatments, based on Fisher’s exact tests, except for variables age, the number of kids, risk attitudes, and time preferences (kwallis). The number of observations varies slightly across questions because some participants did not fill out the complete questionnaire, and not all questions being mandatory

**Table 15:** Gender matches

Matches	Treatments			Total
	*StrangerAdult*	*StrangerChild*	*OwnChild*	
MM	8	13	14	35
FM	27	28	33	88
MF	23	15	16	54
FF	34	36	29	99
Total	92	92	92	276

The table presents the amounts of matches for the observer treatments. The gender of the respective decision-maker is indicated by the first letter and the observer’s gender by the second letter. So, e.g., FM stands for a match with a female decision-maker and a male observer

## Appendix C: Related Literature

### Contextual Changes to Motivate VCA

Bruns et al. (2018) implement a default option to nudge experimental subjects in the lab to contributions to carbon offsetting reductions. Similarly, Araña and León (2013) show that an opt-out condition for VCA programs increases a VCA compared to an opt-in condition. Results from field studies suggest that if supporting a VCA is a pre-set default option, this also increases the average contributions of experts in the field of environmental economics (Löfgren et al. 2012). This effect is stable over longer time periods (Kesternich et al. 2019). Stimuli like matching and rebate subsidies also have positive effects on increasing a VCA (Kesternich et al. 2016). Energy-saving initiatives (such as social norm nudges) have also been found to be effective in creating long-lasting effects on a VCA (Allcott and Rogers 2014; Jachimowicz et al. 2018). A recent study by Böhm et al. (2020) finds that changing the default contribution level as well as providing individuals with the possibility to commit themselves to inter-generational solidarity leads to higher investments into long-term contributions for future generations. Also, Carattini and Blasch (2020) point out that nudges like leveraging social norms can be effective in increasing carbon offsetting behavior. Moreover, Andre et al. (2021) document that informing participants about the true prevalence of climate norms increases climate donations.

### Public Goods and Observability

Past work has shown that mechanisms such as direct and indirect punishment, direct rewarding, as well as reputation building, foster contributions to public goods in the laboratory (see, e.g., Rockenbach and Milinski 2006; Milinski and Rockenbach 2012) and in the field (see, e.g., Balafoutas et al. 2014). Observability in conjunction with punishment (Fehr and Gächter 2000), rewards (see, e.g., Hauser et al. 2016; Rand et al. 2009), communication (Miller et al. 2002; Bracht and Feltovich 2009; Balliet 2010), and framing (Andreoni 1995; Rege and Telle 2004) also positively influence cooperative behavior in the laboratory. Interestingly, when participants can choose, they only make high contributions observable for others (Rockenbach and Milinski 2011). Furthermore, a burgeoning literature using field experiments has shown that being observed, even without the explicit mention or possibility for punishment or reward, also increases cooperative behavior (see, e.g., Bateson et al. 2006; Ekström 2012; Yoeli et al. 2013). The effect is typically stronger in the case of “overt observability”, which means that actual identifying information (e.g., name and face), as well as behavior, are revealed to the observer at or after the point of decision (Bradley et al. 2018).

### Types of Observers

Most existing research has used adults (who are unrelated and strangers to the decision-makers or DMs) as observers. However, for observability to have the largest effect on an intergenerational public good, we argue that a link between today’s DM and the future generation needs to be established. Past research has found that increasing the salience of the beneficiaries of an altruistic decision (the “identifiable victim”) can lead to more giving (Small et al. 2007). Thus, we propose that an observer who directly benefits from the public good, such as a representative of the future generation (e.g., a child today), will be more influential on the DM’s decision than an observer from the current generation (e.g., an adult). In addition, adults who are observed by a child may also want to act as a role model by acting virtuously or in line with societal expectations (Adriani et al. 2018).

### Genetic Link to the Future

The effect of an observer can be further increased by choosing a particularly relevant representative of the next generation—specifically, a parent’s own child (e.g., Ben-Ner et al. 2017). We expect one’s own offspring to be important, as parents have a vested genetic interest in their children (Hamilton 1964a, b; Trivers 1972; Rand and Nowak 2013) who benefit from the VCA.

This genetic connection can prompt other motivations in parents to act in positive ways in front of their children. For example, parents typically want to impart knowledge and good decision-making to their children (see, e.g., Ben-Ner et al. 2017) and be viewed as role models by their own children (see, e.g., Knafo and Schwartz 2001). Indeed, children are influenced by the behavior of their parents when it comes to criminal behavior (McCord and McCord 1958), educational choices (Dryler 1998), and career development (Keller and Whiston 2008).

Furthermore, there is evidence that knowledge and attitudes with respect to climate change are exchanged between parents and children (Lawson et al. 2018). For example, Lawson et al. (2019) find that parents become more concerned about climate change when this issue is brought to them and discussed by their children. Parents are even more likely to engage in actions that benefit their offspring, compared to a situation where they themselves would benefit (Cassar et al. 2016). Observation by their child will, therefore, most likely trigger the parent’s investment in VCA, relative to other observers, as the genetic link to the future is most salient in *OwnChild*.

It is worth pointing out that *OwnChild* combines the individual components of all treatments relative to *NoObserver—*i.e., (i) have DMs be observed, (ii) by a representative of a future generation, (iii) to whom the DM has a genetic link to. Thus, testing if participants’ VCA behavior is highest when the observer is the participant’s own child, is smaller when the decision is observed by a stranger child, further decreases when the observer is an adult observer, and is lowest for the conditions without an observer not only contributes to our understanding of the role of the genetic link in VCA but helps inform policy: if *OwnChild* has a significant effect over *NoObserver*, a social planner would benefit from a policy intervention that meets (i)–(iii). If, on the other hand, both *OwnChild* and *StrangerChild* are significant relative to *NoObserver*, only (i) and (ii) need to be fulfilled, and if all three conditions are significantly different from *NoObserver*, only (i) needs to be met.

By examining and comparing the treatment variations in detail, we can delineate further what drives the effect. In both *OwnChild* and *StrangerChild*, the observer is a representative of the future generation, but only in the *OwnChild* condition, the parent has a genetic link to the observer. Thus, the salience of the genetic link to the future should be higher in *OwnChild*.

While traditional observability studies commonly use adults as observers (e.g., Hoffman et al. 1996), both the *OwnChild* and *StrangerChild* conditions use children who are representatives of future generations. In contrast, the *StrangerAdult* condition resembles the more traditional observability condition where an adult observes the decision. If being reminded of future beneficiaries through the presence of a child observer or wanting to act as a role model in front of a child (regardless of whether or not there is a genetic link), plays an important role, we should expect the treatments where children are watching to yield larger effects than adult observers.

Across all observability treatments, an observer—child or adult—is present to watch the decision-maker relative to the *NoObserver* condition. Based on past literature (see Bradley et al. 2018 for a review article on observability), DMs would be expected to invest more in VCA if being observed, compared to not being observed. Therefore, we compare all conditions with an observer combined (*OwnChild*, *StrangerChild,* and *StrangerAdult*) to the *NoObserver* condition.

## Appendix D: Sample Size and Power Calculation

Our study aimed to create a realistic and meaningful trade-off for participants, who were provided with an endowment that they needed to allocate between themselves and planting trees for the future. We chose an endowment of 69€ (or, equivalently, 46 trees at 1.50€ each, which implies offsetting up to 82.8 tCO2 over 120 years) per participant as a meaningful amount for this trade-off.

The target sample size was 400 parents (100 per treatment), and we aimed for at least 300 parents (75 per treatment). These numbers were based on power calculations, presented in detail in the next section. Also, this ceiling was imposed because recruiting eligible participants for this lab-in-field experiment was not without challenges, as it requires enlisting parents with a child in the pre-determined age range for all conditions in a public space. Based on our experience in running lab-in-field experiments, we were confident that we could recruit up to 400 participants that fit this description. Originally, depending on the recruitment progress by the end of 2020, we planned to adjust our expectations and stop at 75 participants per treatment (to ensure we were able to stay within the time limits of the grant funding we received for this study). However, because of the Covid-19 crisis beginning to show effects in Austria by February 2020, we had a hard stop imposed by the pandemic, leading to 368 participants (92 by treatment) in our final sample.

### Parameters for Power Calculation.

In the following power calculations, we made few, highly conservative assumptions to ensure we are well powered for our study. To inform our power calculations, we conducted a literature review to identify a closely related experimental design. We identified a recent paper by Bruns et al. (2018), who gave participants an endowment of 10€ to allocate between themselves and a “climate protection fund” (CPF), which was later used to purchase carbon licenses from the EU Emission Trading Scheme. Although the CPF is not identical to planting trees, we believed the trade-off between personal enrichment and their chosen VCA is relevant for our study.

The aim of Bruns et al. (2018) was to test different nudges—including information provisioning and shifting the default—to encourage investments into the CPF. In their study, participants in the control condition invested, on average, 18.2% (sd = 26.6) of their endowment into the CPF (technically, 1.82€ out of 10€). Across their treatment variations, the average mean investment was generally uniformly higher than the control group (with values ranging between 28.5 and 30.4% of the endowment invested). For the purposes of our power calculation, we chose the lower bound of these estimates (mean = 28.5%, sd = 29.5). Note the largest standard deviations in both the control and treatment estimates suggest large variability in how participants choose to invest (or not invest) in the CPF. Based on the values in Bruns et al. (2018), we calculate Cohen’s d, a standardized measure of effect size commonly used in the literature: *d* = 0.367.

### Statistical Test and Statistical Power

We planned to use two-tailed Wilcoxon-Mann–Whitney (WMW) tests, a statistical test that makes no assumptions about the distribution of underlying data and is also suitable for any non-parametric distribution. The WMW is a more conservative choice relative to standard parametric tests. We vary the statistical power for the WMW between 80 and 95% to illustrate the implications on the sample size.

We want to emphasize that the assumptions above are highly conservative (e.g., the use of a non-parametric test, powering for the smallest anticipated effect size based on only two conditions). As such, we interpreted the calculated sample sizes as the *upper bound* of what we expected to need. Doing the sample calculation, we kept in mind that, in reality, the effect size may be larger due to parents being generally more generous/engaged when their offspring is observing (see Section Appendix C for the related literature). Also, we considered that our assumptions might have been overly conservative (e.g., that the effect size is the same size for all tests when, in reality, it is likely that the effect size is larger between, for example, our combined treatments and the control group).

Our power calculations hinged drastically on the size of the expected treatment effect. Assuming a similar effect size as Bruns et al. (2018), we assumed to require between 124 and 204 participants per condition. The lowest sample size estimated (*N* = 124 participants at 80% power) would have allowed us to test three out of four conditions, whereas we would only be able to meet the highest sample size estimate (*N* = 204 participants at 95% power) if we reduce the number of conditions to two (*Stranger Child* versus *Own Child*).

However, we anticipated that our treatment is more effective (for example, an increase in Cohen’s *d* by about 1/3) than the nudges in Bruns et al. (2018). This can be assumed because having the parent’s own child as an observer increases the observability effect (e.g., Ben-Ner et al. 2017), as parents have a vested genetic interest in their children (Hamilton 1964a, b; Trivers 1972; Rand and Nowak 2013) who benefit from the VCA. Moreover, as shown by Lawson et al. (2019), parents become more concerned about climate change when this issue is brought to them and discussed by their children.

Following, we expected to be well powered, having 100 participants in each treatment condition. This implied that, at the lowest and middle sample size estimations (*N* = 70 at 80% power and *N* = 93 at 90% power), we would have been sufficiently powered for all conditions with equal sample sizes, while at the highest sample size estimation (*N* = 115 participants at 95% power), we would have been close.

### Pre-Registration

Based on the power calculation, we pre-registered our experiment using aspredicted.org. Please find the detailed pre-registration here: https://aspredicted.org/gt8md.pdf

## Appendix E: Details Experimental Procedure

### Recruitment Protocol

Participants were recruited to our study via public recruitment stands. These were generally presented as part of the University of Innsbruck, offering participants to take part in a paid research study without giving away the purpose of the study. Whenever an adult with at least one child passed by the recruitment stand, they were invited to participate in the study. We asked upfront what the age of the child/children is/are to ensure that at least one child was in our pre-defined age range (7–14 years). Whenever two parents were willing to participate, we randomly chose one parent.

The participating parent was randomly assigned to one of our treatments. To ensure that all participants received the same information, we followed a standardized verbal script described in the section below. If the parent was assigned to the *OwnChild* treatment, we asked that the child “assisted” us as the “helper” by observing the parent’s decision and filling it into a handheld form. If more than one accompanying child was between 7 and 14 years, we randomly chose one child and asked if s/he assists the parent. If the parent took part in one of the stranger treatments, we randomly chose either the confederate child or adult as the “helper.” Participants only knew that the decision involved a potential for earning money, and we emphasized to everyone that all data collection was anonymous and would only be used for this study.

In case a parent took part in one of the three observer treatments, the observer was instructed to act as a “helper,” whose task is to document the decision they make. The helper was given a paper form and was instructed to document the decision on this form. Moreover, all helpers were always carefully instructed that the parent must make the decision on her/his own. This meant that we made sure that the helper (including a parent’s own child in the *OwnChild* condition) did not influence the decision-maker. At the end of the study, participants were paid in cash (for any amount that they chose to keep during the study) and received a certificate documenting how many trees they planted (indicating the number of trees they chose to plant).

### Verbal Experimental Instructions

Please note that the experiment was conducted in German. The following is a translation.

[General instructions at the start of participation for all treatments.]

Welcome to the study, and thank you for participating! Today, you will be asked to make a decision. This decision is payoff-relevant. This means that depending on how you decide, you will receive more or less money (cash) in the end. To show you that we are not joking, here you have an envelope, with the maximum possible payment for today, which is 69€. I will leave the envelope right in front of you during the experiment so that you know and see that your decision is about real money. Only positive monetary payments are possible for your payment today. Please also note when you make the decision that there is no right or wrong. Just decide based on your gut feeling.

Everything today is anonymous. This means that you really make whatever decision you want, as nobody will know who you are.

For the study, you will find all instructions on the screen. If you have any questions, please raise your hand, and we will come to you and help.

[General additional instructions, for all treatments with observers].

We have here some helpers today from the community to support us. Your helper will assist you in answering some questions at the beginning. Afterward, there will be a point where s/he will document your decision. It is essential that you make your decision on your own, so decide yourself. This means you should not discuss with your helper what decision you should make. This is really important. After you made your decision, your helper will bring us a form where s/he documented your decision. You will then be asked to fill out a short questionnaire. When you finished the questionnaire, please raise your hand.

[General additional instructions, for OwnChild].

After your child brings us the paper form, we will take care of him/her outside in the waiting area. S/he will be supervised by us until you finished the study.

[After the verbal instructions, we started the oTree program on the participant’s pc..]

### Digital Experimental Instructions

Screenshots of the experimental instruction provided on the computer screen are available on OSF (see https://osf.io/2kdgz/?view_only=118e3af382284948835e8e66b4d1f451). We indicate if a screen was different for one treatment. Please note that the instructions were translated from German to English. German instructions are available on request.

## Appendix F: The Foresting Program “For the Climate—Tree by Tree”

As mentioned in the main text, we specifically set up a local foresting program in collaboration with the “Amt für Wald und Natur” of the city of Innsbruck (Austria) to help realize the impact and implementation of this project. The trees financed by this program were planted in 2020 and 2021 on the “Nordkette” and “Patscherkofel” mountain ranges close to Innsbruck (see Fig. 4).

Moreover, the city council of Innsbruck was particularly pleased by this project, and they decided to dedicate an entire geographical area to the plantation of the project’s trees, which is now known as the “StadtKlimaWald” (which translates to “city climate forest”). This was decided and implemented after all data was collected and therefore could not affect the participants’ decision process. The StadtKlimaWald is located in the city area called “Burgstadl” of Innsbruck (see Fig. 5).

We are delighted that our project produced this additional, long-term added value with the StadtKlimaWald. It is barrier-free accessible for everyone and has become a highly frequented area for people from Innsbruck of all ages. Moreover, informational material is provided at one of the sites of the forest to inform the public about the importance of mixed-tree forests for the climate. More information can be found on the website www.baumfuerbaum.com (only available in German).

## Abbreviations

DM: Decision maker
VCA: Voluntary climate actions

## Footnotes

1 We thank an anonymous reviewer for this observation and the suggested analysis. In addition to the simple correlation between the two variables, we also explored whether including both in our standard regression framework would alter the results. When both a dummy variable for education and a continuous variable for the climate change perception index is included in the regression, both covariates are both independently significant, but they have no effect on our main results: our treatment effects are robust to their inclusion (results not shown).

2 The literature typically calculates willingness to pay (WTP) for offsetting one ton of CO2. We cannot calculate the WTP for this quantity, as the cost of offsetting one ton of CO2 is fixed by design in our experiment (100€ per ton). Instead, we have calculated the WTP for offsetting some amount of a ton of CO2 (i.e. the number of trees planted, each of which offsets CO2 by some fraction each year).
